# Utility of early diagnosis, contact tracing and stakeholder engagement in outbreak response in three COVID-19 outbreak settings in Ghana

**DOI:** 10.4314/gmj.v55i2s.5

**Published:** 2021-06

**Authors:** Mary Amoakoh-Coleman, Delia A Bandoh, Charles L Noora, Holy Alomatu, Abraham Baidoo, Sally Quartey, Ernest Kenu, Kwadwo A Koram

**Affiliations:** 1 Department of Epidemiology, Noguchi Memorial Institute for Medical Research, University of Ghana, Legon, Accra, Ghana; 2 GFELTP, Department of Epidemiology and Disease Control, School of Public Health, University of Ghana, Legon, Accra, Ghana; 3 Tema Metro Health Directorate, Ghana Health Service, Tema, Ghana

**Keywords:** contact tracing, stakeholders, laboratory, early diagnosis, COVID-19

## Abstract

**Objective:**

To describe how early case detection, testing and contact tracing measures were deployed by stakeholders in response to the COVID-19 outbreak in Ghana – using three outbreak scenarios.

**Design:**

A descriptive assessment of three case studies of COVID-19 outbreaks within three settings that occurred in Ghana from March 13 till the end of June 2020.

**Setting:**

A construction camp, a factory and a training institution in Ghana.

**Participants:**

Staff of a construction camp, a factory, workers and students of a training institution.

**Interventions:**

We described and compared the three COVID-19 outbreak scenarios in Ghana, highlighting identification and diagnosis of cases, testing, contact tracing and stakeholder engagement for each scenario. We also outlined the challenges and lessons learnt in the management of these scenarios.

**Main outcome measures:**

Approach used for diagnosis, testing, contact tracing and stakeholder engagement.

**Results:**

Index cases of the training institution and construction camp were screened the same day of reporting symptoms, whiles the factory index case required a second visit before the screening. All index cases were tested with RT-PCR. The training institution followed and tested all contacts, and an enhanced contact tracing approach was conducted for staff of the other two sites. Multi-sectorial engagement and collaboration with stakeholders enabled effective handling of the outbreak response in all sites.

**Conclusion:**

Comparing all three settings, early diagnosis and prompt actions taken through multi-sectorial collaborations played a major role in controlling the outbreak. Engaging stakeholders in the COVID-19 response is an effective way to mitigate the challenges in responding to the pandemic.

**Funding:**

The COVID-19 outbreak response and writing workshop by the Ghana Field Epidemiology and Laboratory Training Programme (GFELTP) was supported with funding from President Malaria Initiative – CDC, and Korea International Cooperation Agency (on CDC CoAg 6NU2GGH001876) through AFENET

## Introduction

Since the novel coronavirus 2019 (COVID -19), was identified in Wuhan City in the Hubei Province of China in December 2019^1^ the virus has caused significant morbidity and mortality as it rapidly spread throughout the world. 2 COVID-19 infections was declared a global pandemic by the WHO on March 11, 2020. By the end of June, 2020, it had infected over 10 million persons across 213 countries and territories, with about 120,000 fatalities recorded.^2^

Ghana recorded its first case on March 12, 2020 and total cases and deaths stood at over 17,000 and 100 respectively by the end of June 2020. 2,3

Despite current limitations in knowledge of COVID_19 disease transmission, the disease certainly spreads from person to person, from respiratory droplets of both symptomatic and asymptomatic infected people, commonly through coughing and sneezing. Early diagnosis, complete contact listing, timely contact tracing and prompt isolation of cases are key to preventing transmission of outbreaks such as COVID-19. 4–6

These measures help control sporadic cases and clusters and prevent community transmission through effective mechanisms for quarantine, isolation, treatment and monitoring of contacts and cases, and suppression of community infection transmission through context-appropriate measures.^6^

Outbreak responses, including early diagnosis and contact tracing, affect the impact of outbreaks. Outbreaks usually affect individuals and communities. Therefore, an effective response involves both the community and other stakeholders.^8^–^10^ Stakeholders in outbreak response refer to individuals and groups who form part of the affected population or might be affected by the effect of the outbreak and are interested in the response process thus are willing to take ownership of it in their setting.^8^,^10^

Stakeholders in an outbreak become the link between the affected population and the response team. Their role also involves communicating the outlook of the outbreak to the population and demystifying rumours and fears due to the outbreak.^9^,^11^,^12^ Due to the key role of stakeholder collaboration, Ghana's COVID-19 outbreak response brought together the Ghana Health Service, Ghana Field Epidemiology and Laboratory Training Programme (GFELTP) and stakeholders from various local settings to assist in early diagnosis and management of the outbreak.

Ghana developed documents for early identification of cases, prompt contact tracing and case management. These protocols were used in the training of health workers and health education. National laboratories were also equipped to test the virus using Reverse-Transcription Polymerase Chain Reaction (RT-PCR) tests. Health workers were trained for contact tracing of cases and data management systems that allow for tracking and decision-making. These measures evolved in different locations based on the spread, with Accra and Kumasi being the initial points of the outbreak.

Similarly, the implementation of the protocols was based on specific considerations and the dynamics of the affected populations. Regular evaluation of all outbreak response strategies is important, particularly early diagnosis and contact tracing as the outbreak spreads. We examined how stakeholders deployed early case diagnosis and contact tracing measures during Ghana's COVID-19 response in three different settings.

## Methods

### Design

A descriptive assessment of three case studies of Ghana's early COVID-19 outbreaks in three settings from March 13 to June 30 2020.

### Setting

Three settings in Ghana, a country located in West Africa with 30 million people, were studied. Ghana recorded its first two cases of COVID-19 on March 12, 2020. Subsequently, the number of cases increased to over 17,000 as of the end of June 2020.

The first of the three settings was a training institution in the Greater Accra Region, one of the largest in the country, and located within a district identified as a hotspot during the first two months of the outbreak. The institution has a population of over 20,000, consisting of staff and students. Facilities include academic faculties, residential facilities, commercial and recreational centres, and a hospital that serves both the institution and the district.

The second set was a construction camp located in the Eastern Region of Ghana. The camp houses over 200 workers of a railway construction firm and is located in an isolated community, surrounded by small rural settlements. The workers share rooms, living areas and toilet facilities. They are served meals in a common dining area in batches and have recreational grounds for sports activities. Shuttle services are available to and from the construction sites and living quarters. The camp has a health post operated by a physician assistant who attends to minor ailments of workers.

The third setting was a food processing factory located in a metropolitan area close to Ghana's capital. It has about 2,000 workers, including casual workers, who reside either within the metropolis or in the neighbouring districts. The factory provides shuttle services for workers to and from work, and a staff canteen serves them food. Minor ailments and injuries are managed at an onsite clinic managed by nurses and laboratory personnel under the supervision of an institutional physician.

### Process description

We described and compared three COVID-19 outbreak response scenarios in Ghana. Our description highlights identification and diagnosis of cases, testing, contact tracing and other containment measures and stakeholder engagement for each scenario. We also outlined overall challenges and lessons learnt in the management of these scenarios.

### Data sources

We reviewed daily situational reports, interim and final reports and all documents about the outbreak in these three settings. These included community/ institutional reports, field epidemiology teams' reports, and reports from the health directorates.

Relevant data were extracted from the routine COVID-19 surveillance data management system of the Ghana Health Service into Microsoft Excel for the three sites, cleaned and analysed descriptively and presented as a table.

### Ethical consideration

We obtained ethical clearance from the Ghana Health Service Ethics review committee (GHS-ERC 006/05/20). The Ghana Health Service (at district, regional and national levels) and the institutions concerned provided and gave permissions for the data to be used. Participants were assured of the confidentiality of the information shared. All information obtained was stored on password-protected computers.

## Results

### Identification and diagnosis of index cases

On March 13 2020, a day after Ghana recorded its first case of COVID-19, a 36-year old female student in a training institution who had returned from a trip abroad a week earlier reported to a private facility with a history of fever, cough, general weakness, runny nose, headache. Persons with a recent travel history to a country with reported cases of COVID-19, COVID-19 was suspected, a sample was taken for testing, and a diagnosis of COVID-19 was made on March 14, 2020. The case was isolated for management. A total of 96 contacts within and outside the campus were listed. Of these, 74 were students, and 72 of them underwent 14-days of mandatory quarantine on campus. Three of the quarantined contacts developed some mild cough, cold, and fever symptoms during the follow-up follow-up period, but all three tested negative for COVID-19. There were no major symptoms reported among the remaining contacts during this period. At the end of the 14-day quarantine period, 94 contacts tested negative and were discharged.

The index case at the construction camp in the Eastern region was a 32-year-old male worker with a railway construction company. He reported to the camp's health post on March 30 2020, with a cough, fever and a recent history of travel outside the country. As the first suspected case reported in the region, immediate attention was given at all levels. The health directorate was informed, and samples were taken on March 30 2020, for testing. Results came out positive for COVID-19 on March 31 2020. The case was taken to the isolation centre for management. Subsequently, all 249 workers in the camp were listed as contacts due to the nature of the camp and the level of interaction among its occupants. Contacts were quarantined for 14 days and tested. In the following week, 15 new cases were recorded from the contacts tested.

Testing of contacts continued, and the number of cases eventually increased to 69 by the third week. Identified contacts and asymptomatic cases were quarantined or isolated at the camp or designated quarantine/ isolation centres in the region. Daily charting of symptoms and signs was done for all cases and retested after 14 days. A case had to test negative twice before being discharged from the isolation centre. All confirmed cases were asymptomatic, and no death was recorded among them.

The index case at the factory was a 48-year-old female worker who reported to the company's clinic on April 9, 2020, with cough, fever, chills, and body pains. Two weeks before presentation, she had been seen with symptoms of cough and unwellness and had been given some medications and discharged. On this second occasion, with a high index of suspicion of Covid-19, a sputum sample was taken on April 9, 2020, and tested. The positive result for COVID-19 was received on April 17, 2020. The case was taken to a designated isolation centre for management. Identified contacts of the index case at work were listed (160 workers) and tested while other employees of the company agreed to undergo voluntary testing (enhanced screening) for COVID-19 (additional 978 workers). Out of 1,138 employees whose samples were taken, 695 positive cases were recorded for COVID-19, 128. employees tested negative, while 315 did not receive their test results for more than three weeks. Case management teams were formed to handle disclosure of results to confirmed cases (most of whom were asymptomatic and managed at home), management plans and risk communication to primary contacts.

### Testing

The three case scenarios occurred early on during the outbreak in Ghana. Depending on the availability of test kits, nasopharyngeal, or oropharyngeal or sputum samples were taken. For all index cases, samples were taken immediately at the point of care and analysed using RT-PCR at the Virology Department of the Noguchi Memorial Institute for Medical Research (NMIMR).

Testing for identified contacts for the construction camp case and the training institution also took place at NMIMR, with samples taken by Ghana Health Service (GHS) and the institutional health facility staff. However, the factory and construction camp advocated for and eventually had mass testing for many of its staff because they were concerned that routine daily interactions potentially would have brought the case into contact with a wider population of the staff. For factory contacts, some had their tests analysed at NMIMR while others were done at the Public Health Reference Laboratory (PHRL) of the GHS because of their large numbers and to avoid delays in getting results.

The positivity rate after the initial testing was 61.1% (695/1,138). This resulted in many staff undergoing isolation, with a few (9) refusing to be isolated because they did not believe the results were truly positive. Turnaround time for test results for all index cases was two days but varied considerably for the tests for contacts. It was on average four days, ten days and two days for the construction camp, factory and training institution.

Some factory contacts, especially on retesting, did not get their results and were eventually declared discharged when they remained asymptomatic after 14 days based on the revised national protocol. Testing was at no cost to cases and contacts by the national testing policy. Table 1 shows a summary of some key features of the cases and contacts.

**Table 1:** Summary of cases and contacts for three case study populations

Community			Index case		Total contacts of cases identified	Positive contacts		Deaths*
	Sex	Age	International Travel History	Known contact of a case		Male	Female	
**Construction camp**	Male	32	Yes	No	249	69	0	0
**Factory**	Female	48	No	No	1,138#	345	350	0
**Training Institution**	Female	36	Yes	No	96	0	0	0

* All cases were declared recovered either with two (2) consecutive negative test results or absence of symptoms after 14 days

# Includes all listed and tested after the first case was identified due to the nature of the work

### Contact tracing

The GHS team listed contacts from the airline the case used, household, faculty, and other contact points on campus for the academic institution. Contacts were then identified through phone calls and informed of the 14-day quarantine they were to undergo. Contacts from the airline and household were followed by GHS. At the same time, the GFELTP residents and supervisors were deployed as the contact tracing team responsible for the students and faculty contacts on campus. All the contacts were initially tested for COVID-19 and found to be negative for the infection. Student contacts were quarantined on campus to make follow up easier. This was made possible because the institution was closed as part of the national response to the outbreak. Faculty contacts chose to self-quarantine at home.

All contacts were monitored daily with symptoms charting, given psychological support at least once a week, and provided with food and housekeeping supplies throughout their stay. At the end of the mandatory 14 days quarantine period, all contacts tested negative and were discharged to go home.

The entire camp was quarantined since they were all listed as contacts, with the district surveillance team responsible for contact tracing. The identified contacts to the index case were quarantined for 14 days in the camp. Contacts were tested at the beginning of quarantine. Positive contacts were isolated in the camp's isolation centre set up by the construction firm. This centre was located on one side of the camp, where the movement was prohibited to prevent further contact.

Contacts were monitored daily with symptoms charting for 14 days, tested after the quarantine, and discharged based on two negative test results. For the factory, primary contacts of the index case in her household were listed, identified and quarantined. Contacts were monitored daily with symptoms charting for 14 days, tested and discharged based on two negative test results.

All household contacts tested negative. Contacts in the factory were identified and tested. The factory requested mass testing of all other workers based on the sensitivity of the work it does. Cases from among the staff found were identified, and their contacts were listed through phone interviews. Some cases, however, refused to accept their test results and so did not disclose their contacts. Those living outside the factory district were transferred to their districts of residence for contact tracing and follow up of their contacts. Contacts to cases in other districts were followed up, but only a few districts reported back on them. Contact tracing teams were formed to follow-up contacts of cases living in the factory district. Some contacts refused to cooperate with contact tracers making it difficult for an effective 14 day follow to be done.

### Other containment measures

In addition to early diagnosis and contact tracing, other containment measures were in place in all three scenarios to make the response effective.

Handwashing facilities and some alcohol-based sanitisers were provided at the training institution, at the entrance of the quarantine hostel. A “no mask no entry” policy was instituted to ensure that all contacts and staff were protected. Some detergents and PPEs were provided for workers for cleaning the hostel, and the place was fumigated before and after contacts occupied it. Hand sanitisers were provided for each contact during the period.

For the camp, segregation of workers to different parts or zones based on their infection status (case, contact) enabled the facility to be fumigated. There were training sessions for factory workers on the infection and management and containment protocols and appropriate PPEs. Workers were sensitised on what COVID-19 was, early signs and symptoms to report, how to prevent getting infected, and psychological support, especially for cases.

For the factory, an infectious disease preparedness and response plan was developed. Handwashing facilities comprising running water, soap and hand dryers were provided at the entrances. The temperature of all persons entering the factory, including staff were taken daily. All workers needed to wear facemasks always when within the factory. A “no facemask, no entry” policy was instituted, and signage on the same was displayed around the facility. Floor markings were put in place within the production plant to ensure physical distancing. The number of persons per table at the canteen was reduced.

Appropriate personal protective equipment (PPE) were provided and used by the staff of the factory clinic following training on how to use them. Work-related meetings were conducted virtually. A well-resourced room was designated as a holding centre for staff who may present with suspected symptoms of COVID-19.

### Stakeholder engagement

There was a collaboration between stakeholders in responding to these pockets of outbreaks. For the training institution, the facility authorities promptly notified the GHS, which kicked in the response immediately. Management of the institution was notified, and its emergency response team (ERT) was called to manage the response on the campus, also ably supported by the GFELTP team. The collaborative team worked with the institution management to provide boarding and lodging, education, psychological support, monitoring, testing and eventual discharge. The faculty leadership and school members from where the case index case originated were very supportive in providing sanitary and housekeeping logistics for the contacts while under quarantine on campus.

Contacts to the index case who were living outside the campus were managed by the GHS directly. On average, all measures were instituted in less than a week.

In the construction camp, the camp physician informed the district health directorate and right away, support was received from the regional health directorate to facilitate testing, contact tracing and data management. The human resource department of the construction firm was also pivotal in organising the affected workers throughout the process to undergo procedures, providing resources for infection prevention and control, and observing all protocols to reduce or avoid the spread of the infection in the camp. There were meetings between the facility management and the regional and health directorates to provide education, psychological support and monitoring. A GFELTP resident supported the district with contact tracing and all other containment processes. On average, all measures were instituted in less than a week

At the factory, the management worked with the local government authority, the district health directorate, the regional health directorate and the public health directorate of the GHS. Specifically, The Deputy Director of Public Health (DDPH) for the region and the national Disease Surveillance Department of GHS was duly notified. In turn, they gave guidance to the district to help contain the infection. On their part, the factory made their facility available for test samples from workers and contacts to be taken there so that staff could easily commute to the testing site. Again, the team from GFELTP was very instrumental in providing human resources and other logistics to support testing and contact tracing. As part of the containment measures, the company had to operate at a limited capacity. Factory management had issues with this. This was partly due to delays in getting results from the laboratory to discharge those who potentially may have become negative to return to work. Some results took as long as three weeks. Whiles others were never returned. The team continued to facilitate improvement in turnaround time for test results by engaging the laboratories. Before the full reopening of the factory, a team from GHS, the local government office and field epidemiologists from the GFELTP conducted an assessment of the factory's preparedness for reopening. A report of the assessment was submitted to the regional health directorate and the local government office for approval to reopen.

The team worked to support the factory to be ready to operate in a safe environment where all COVID-19 preventive protocols are in place and adhered to. Management were cooperative in getting all the required measures in place. On average, it took more than a week for measures to be put in place.

## Discussion

We set out to examine how early case diagnosis, testing and contact tracing measures were deployed in response to COVID-19 outbreaks in three different settings in Ghana through collaboration between stakeholders.

Early diagnosis, including testing, contact tracing and stakeholder engagement, proved to be important for managing these three cases of COVID-19 outbreak. Spread was reduced as there were no new cases beyond the primary contacts. Swift and prompt identification of contacts and contact tracing in COVID-19 response is essential due to the novelty of the disease and the new information obtained about it daily.^6^

### Early diagnosis

As a global pandemic, initial COVID-19 cases reported in countries were associated with a history of travel to another country. However, community or vertical transmission becomes inevitable when the country cannot contain imported cases due to the nature of the spread. In the case of the three scenarios described, two had travel history, making it easy to suspect COVID-19 on presentation with symptoms. However, in the case of the factory scenario, the patient had no travel history. Thus, though she presented with symptoms, COVID-19 was suspected only at the second visit, delaying diagnosis. Delay in diagnosis leads to further spread of the disease. There is a need to sensitise health workers on the possibility of community spread a disease or a pandemic starts immediately. This can lead to alertness and a high index of suspicion of cases.

The laboratory plays a major role in responding to outbreaks. In the case of COVID-19, the laboratory's role in diagnosing cases cannot be overemphasised. This is because the standard for diagnosing a suspected case was a positive laboratory test result. In all three cases, laboratory testing of samples was a vital step in managing the situation.

The turnaround time for all three cases scenarios ranged between 2 and 10 days and even in some cases, no results were obtained. There is a strong need to consider laboratory capacity when planning outbreak response strategies. In the factory, mass testing was a step towards curtailing the outbreak. However, due to the laboratory challenges, some results were not received. A longer turnaround time implies the potential spread of the disease since individuals whose samples were taken continue to go about their daily lives while waiting for their results. Lapses of this nature tend to impact the containment of the outbreak negatively. Positive cases never get to know their status, leading to further spread of the disease to their contacts.

Again, suspected cases begin to agitate and feel uneasy waiting for their test results. Delay in response from laboratories and long turnaround time lead to delay in containing outbreaks and difficulty in managing the situation.^13^ If unattended to, diagnostic lapses could result in mistrust in the health system. Building laboratory capacity includes decentralisation of laboratory testing, increasing the number of testing sites, and training more laboratory staff to meet the demands of the people.

Completeness and promptness of contact listing, identification and tracking and tracing of contacts of cases in COVID-19 response help in containing its spread. Ideally, contacts of a case should be identified and contacted 24 hours after case results have been declared.^6^ When there are delays in identifying cases, it can lead to further spread since the required precautions are not observed. In the construction camp and the training institution, all cases, their primary and secondary contacts were identified, listed and followed up for the fourteen-day quarantine period. However, in the case of the factory, the listing of contacts was problematic.

Finding secondary contacts was difficult due to the attitude of some of the primary contacts turned cases. One way to complete the process of identifying, tracking and tracing contacts could be through intense education on the essence of contact identification, quarantine, isolation, and other measures taken after testing. This can improve the willingness of contacts to be traced, quarantined or isolated.

### Stakeholder engagement

Stakeholder engagenment was a major feature in handling the outbreak in all three settings. Multi-sectorial engagement and collaboration with stakeholders is required to deal effectively with contact tracing and outbreak response. There was full support from the institutional management in the institution and at the construction camp while there were some hitches with support in the factory. This explains why contact tracing was effectively done in the two settings and not the latter. Given the role of local authorities in outbreak response, focus must be placed on building their capacity through education, training and constant communication using the right channels.

The multi-sectorial team overseeing an outbreak reponse, needs to be managed daily by experts for an effective outcome.^6^ Though this was done in all three settings, there were hitches in the factory. This could be due to the large number of positives diagnosed at a time and the inability to properly engage all of them to let them know their status.

A major key for mitigating a pandemic is the implementation of a coordinated response which focuses on public health messaging and maintenance of situational awareness^15^. During pandemics like COVID-19, people are confused and disturbed due to conflicting and inappropriate information on various media platforms. Interactions with cases and contacts from all three settings, especially the factory, exposed their fear and uncertainty due to inadequate information. To deal with the pandemic successfully, there is the need to make people feel secure by providing them with accurate information and assurance throughout the period.^15^

Community understanding of COVID-19 is key to getting the exposed population to cooperate with the response team. In all three cases, education of cases, contacts and stakeholders ensured their cooperation throughout the outbreak period. . Approaches such as adopting a single channel of communication to the workers helped them feel secure and helped deal with issues of stigma. Also, engaging professional counsellors and psychologists to talk to cases and contacts allayed their fears

One lesson learnt from the three settings was the effectiveness of protocols in confined settings. All protocols were fully observed in the construction camp and institution where people were camped and could easily be enforced. In a setting where people had no restrictions, enforcement of protocols was challenging. Confined settings were therefore found to help in enforcing protocols to curb the outbreak. A similar situation was seen in China, where strict confinement measures were instituted to help contain the outbreak. The rate of spread was seen to drop drastically.^16^ On the contrary, spread increases exponentially without enforcement of measures.

In addition, approaches used in each of the settings had to be modified based on the institution's set up and way of operation. Each setting has its unique practices, policies and process in place. Again, the educational and socioeconomic status can provide insight into disease transmission within and out of the workplace and further insight into how the outbreak can be successfully handled.^17^ Adaptation of approaches to different settings without considering the setting's dynamics would therefore not yield the required impact. Locally tailored approaches tend to be more effective. 18

Recovery strategies are best adopted based on the location of the outbreak. What worked perfectly in an academic institution may not necessarily work in a factory or a construction camp. Therefore, there is the need to have flexible approaches that are modifiable based on the location, setting, and nature of place the outbreak.

From the three scenarios, the need for an emergency response plan and a team to implement it to be in place before the outbreak occurs is highlighted. Periodic simulation exercises can be done to test the readiness and capacity of the institution to deal with the situation. This is in line with the WHO policy for getting workplaces prepared in the face of COVID-19.^19^

Tackling epidemics in this 21st century requires a multisectorial approach that incorporates the whole society. This is because, gradually, many diverse factors, including social, medical and economic are drivers of epidemics.^20^

### Lessons learnt

- Prompt identification of cases leads to an early response with appropriate measures to curtail an outbreak.
- Long turnaround time for test results negatively impact outbreak containment measures.
- Laboratory capacity needs to be critically considered in situations of mass testing, with consideration
- Prompt and sustained stakeholder engagement and cooperation are important for quick response to the outbreak situation.
- Enforcement of restrictions and adherence to preventive protocols are essential to the containment of the outbreak
- In every outbreak, a clear communication channel regarded by all stakeholders is essential for risk communication.
- An understanding of the context-specific issues in the application of epidemiological systems and protocols is very important for successful outbreak management

One limitation of the study is possible recall bias as field notes were used, and in the heat of the outbreak, it is possible some facts were missed. We contacted stakeholders in the various settings for clarification and additional information during this write-up to address the gaps.

## Conclusion

Comparing all three settings, early diagnosis and prompt actions taken through multi-sectorial collaborations played a major role in controlling the outbreak. Early diagnosis, including prompt testing and release of results, identification, tracing and following up of cases and contacts, is essential in mitigating outbreaks. While there could be challenges due to the extent of the outbreak, the multi-sectoral stakeholder engagement approach involving cases, contacts, their communities, management of institutions and health workers is key to effective implementation of all outbreak response activities.
